# Equine herpesvirus 2 (EHV-2) infection in thoroughbred horses in Argentina

**DOI:** 10.1186/1746-6148-1-9

**Published:** 2005-11-09

**Authors:** María I Craig, María E Barrandeguy, Fernando M Fernández

**Affiliations:** 1 Instituto de Virología, Centro de Investigación en Ciencias Veterinarias y Agronómicas (CICVyA), INTA, CC 25, (1712) Castelar, Buenos Aires, Argentina

## Abstract

**Background:**

Equine herpesvirus 2 is a gamma-herpesvirus that infects horses worldwide. Although EHV-2 has been implicated in immunosuppression in foals, upper respiratory tract disease, conjunctivitis, general malaise and poor performance, its precise role as a pathogen remains uncertain. The purpose of the present study was to analyse the incidence of EHV-2 in an Argentinean horse population and correlate it with age and clinical status of the animals.

**Results:**

A serological study on 153 thoroughbred racing horses confirmed the presence of EHV-2 in the Argentinean equine population. A virus neutralization test showed a total of 79.7 % animals were sero-positive for EHV-2. An increase in antibodies titre with age as well as infection at earlier ages were observed.

EHV-2 was isolated from 2 out of 22 nasal swabs from horses showing respiratory symptoms. The virus grew slowly and showed characteristic cytopathic effect after several blind passages on RK13 cells. The identity of the isolates was confirmed by nested PCR and restriction enzyme assay (REA).

**Conclusion:**

This is the first report on the presence of EHV-2 in Argentina and adds new data to the virus distribution map. Though EHV-2 was isolated from foals showing respiratory symptoms, further studies are needed to unequivocally associate this virus with clinical symptoms.

## Background

Equine herpesvirus 2 (EHV-2) is a slowly growing, cell-associated gamma-herpesvirus. This virus is widespread throughout the equine population and has been isolated from horses of different countries like United Kingdom [1], Japan [2], Australia [3], New Zealand [4], Switzerland [5], Germany [6,7], United States [8,9], Canada [10], Hungary [11] and more recently, from Poland [12]. Although its role as a pathogen is controversial, some authors have reported its association with upper respiratory tract disease, inappetance, lymphadenopathy, immunosuppression, keratoconjunctivitis, general malaise and poor performance [8,13,7,16].

Equine Influenza Virus and Equine Herpesvirus 4 (EHV-4) are the most common viral agents related to respiratory disease in Argentina (Dr. Barrandeguy, personal communication). These and other respiratory viruses as Adenovirus, Equine herpesvirus 1 (EHV-1), Arteritis Virus and Rhinovirus, are usually checked in the diagnostic routine of our laboratory but no records about EHV-2 isolation or frequency of sero-positive samples were available.

The purpose of the present study was to analyse the presence of EHV-2 in an Argentinean horse population and to correlate its incidence with age and presence of respiratory symptoms.

## Results and discussion

Sero-prevalence of EHV-2 was calculated on one hundred and fifty-three (153) thoroughbred racing horses by a neutralization test (NT). Cross reactivity with EHV-5 was not checked in this study. The percentage of sero-prevalence to EHV-2 was 79.7% (122/153). Sera samples were grouped according to the clinical status into animals with symptoms (fever, cough, nasal discharge) or clinically healthy. Again, each of these groups was divided according to the animal age in older or younger than 1 year old. The arithmetical mean of the antibody titres was calculated for each of these four groups. Mean antibody titres between older and younger than 1 year old animals both, with and without clinical symptoms were statistically compared. Mean values for the older horses (1.28 > 1.02) were significantly (p < 0.05) higher than for the younger ones. These results agree with the observations of other authors [17] about the increase in antibodies titre with age. Within the older than 1-year group, the mean titre in the group with clinical symptoms was higher (1.34>1.22) though not significant. However, mean titre values were significantly (p < 0.05) higher (1.19>0.85) in the group with clinical symptoms within the younger than 1 year group (Table 1). This difference might be related to the early exposure to this agent.

**Table 1:** Distribution of serum samples according to age and clinical status

	**With symptoms**		**Antibodies**	**Clinically healthy**		**Antibodies**
**Animals**	Sero-negative	Sero-positive	(Mean titre)^a^	Sero-negative	Sero-positive	(Mean titre)^a^

**Older than 1 year old**	5	32	1.34	3	40	1.22
**Younger than 1 year old**	3	26	1.19	18	26	0.85*
**Total (n)**	66			87		

n: number of samples

a: arithmetical media of Ab titre (Reed and Muench)

*: Significantly different (p < 0.05)

Our results suggest the virus is circulating with a high prevalence on the analysed equine population, in accordance with other sero-prevalence data [17,18], and confirm previous reports [19] about the acquisition of EHV-2 at earlier ages.

Taking into account the relatively high percentage of sero-prevalence and the association of EHV-2 with respiratory disease [16], the isolation of this viral agent from horses showing different respiratory symptomathology was carries out. Twenty-two (22) nasal swabs from horses, aged between 6 months and 2 years old, displaying respiratory symptoms were checked for the respiratory viruses commonly analysed in the laboratory routine, and none of them resulted positive for these viruses. Only two (2) nasal swab samples, named E1 and E2, showed CPE after the third blind passage. Some authors reported the presence of vacuoles in RK-13 cells infected with EHV-2 isolates [20] while others described various CPE forms depending on the virus isolate and cell type [21]. In our isolate, CPE was characterized by rounded cells, syncytia and vacuolized cell aggregates with suited partial cell membrane fusion. The late development of CPE referred to a slow growing viral agent.

In order to identify these two isolates, two simple techniques like EM and IIFA were first used. The particle morphology observed by EM showed a size corresponding to a typical herpesvirus (data not shown). The IIFA showed fluorescence signals on cells infected with EHV-2 reference strain LK, E1 and E2. Mock-infected cells were negative (data not shown). As the polyclonal antiserum used for the staining could have cross reaction with EHV-5, another member of the gamma-herpesvirinae subfamily that shows growth characteristics similar to EHV-2 [22], a virus specific nested PCR and a restriction enzyme analysis (REA) were performed.

**Figure 1:**
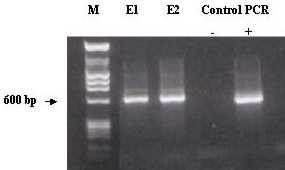
**Nested PCR on nasal swab samples from foals with respiratory symptoms. **Nested PCR amplification products from two equines (E1 and E2) showing respiratory symptoms were analysed by gel electrophoresis. DNA was extracted from original nasal swabs; DNA from the suspension was used as template in the first round. Genomic DNA from EHV-2 strain LK was used as a PCR positive control (PCR+) and H_2_0 as negative control (PCR -). DNA marker φX-174 *Hae *and λ-RF/*Hind *III is seen in lane M.

Nested PCR was carried out on E1 and E2 samples as described in Methods. After the second round, both isolations showed a band of around 600 bp (Figure 1), as the one displayed by the positive control.

**Figure 2:**
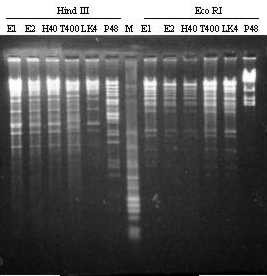
**Restriction enzyme assay profile of EHV-2 isolates and reference strains. **EHV-2 isolates (E1 and E2), EHV-2 strains H40, T400, and LK4, and EHV-5 strain P48, were amplified in RK-13 cell monolayer. Viral DNA was purified by Genomic-tip 20/G column, digested with *Hind *III and *Eco *RI and analysed by gel electrophoresis. DNA marker φX-174 *Hae *III and λ-RF/*Hind *III is seen in lane M.

EHV-5 identity of the isolations was unequivocally excluded by REA. As shown in Figure 2, the patterns after digestion with *Hind *III and *Eco *RI of the isolates and a control EHV-5 were completely different.

As it was specified by others authors [23,24], EHV-2 was described as a virus with a high genomic variation. In our experiments, the restriction patterns of the isolates were different to those of T400 and LK4. Moreover, E1 and E2 resulted identical to each other and very similar to strain H40. Both, E1 and E2 belonged to the same stud farm and were probably epidemiologically related.

## Conclusion

Although EHV-2 has been reported in equine populations worldwide, no reports about its prevalence in Argentina were available. In this study, serological data and virus isolation provided a clear evidence for the presence of this agent on the studied horse population. In horses younger than 1 year old but not in the older ones, serological data correlated with the presence of clinical symptoms. Though viral agents associated with respiratory disease were discarded, no bacterial analysis was done in the studied samples. Further studies are necessary to correlate EHV-2 with the respiratory disease. The EHV-2 identity of the isolated viruses was finally confirmed by the use of a nested PCR and a restriction enzyme assay. This is the first report of EHV-2 isolation in Argentina and adds new data to the EHV-2 distribution map.

## Methods

### Cells

RK-13 (ATTCC CCL 37) cell monolayers were grown with Eagle 's- minimum essential medium (MEM-E) supplemented with 100 UI/ml penicillin, 60 μg/ml streptomycin, 50 μg/ml gentamycin and 10% FCS. Subsequently, the cells were maintained with MEM-E supplemented with same concentrations of antibiotics and 2% FCS.

### Virus

EHV-2 strains LK (ATTCC VR 701), H40 (NVSL Ames Iowa), LK4 and T400 as well as, EHV-5 strain P48, were kindly supplied by Dr. Borchers (Institut für Virologie, Freie Universität, Berlin-Germany).

### Samples

#### Sera

Serum samples were obtained from one hundred and fifty three (153) thoroughbred racing horses, aged between 6 months and 2 years old.

#### Nasal swabs

Nasal swabs from twenty-two horses showing respiratory symptoms like nasal discharge, cough and fever, were used in our research. Samples were collected with a cotton swab and transferred to a tube containing 3 ml of E-MEM supplemented with 2% FCS and antibiotics. Clarification was performed by centrifugation at 3000 rpm (Hermle Z 513 K) for 15 min. Each sample was aliquoted and stored at -70°C until use.

### Neutralization test

This assay was performed on 96 wells RK-13 cell monolayer, where serial four-fold dilutions of complement-inactivated serum were incubated with 100 ID_50 _of LK strain for 4 hs at 37°C as described elsewhere [25]. After 7 incubation days, antibody titres were calculated by Reed and Muench formula [26]. Sera that protected 100% of the cell monolayer at the lower dilution assayed were considered positive. An EHV-2 positive horse reference serum used as positive control, was kindly supplied by Dr. Borchers.

### Indirect immunofluorescence assay

Trypsinised virus infected cells were seeded on IFA micro slides and fixed with ice-cold acetone for 30 min. A polyclonal EHV-2 specific rabbit serum (341 EDV 8301, NVSL Ames Iowa) was added and incubated in a moist chamber al 37°C for 1 h. Fluorescein conjugated rabbit antiserum (KPL Kikergaard and Perry laboratories) was used to reveal the reaction. EHV-2 LK strain infected RK-13 and non-infected cells were used as positive and negative assay controls, respectively.

### Electron microscopy

Cell cultures showing 70% CPE were trypsinised and sonicated for 30 seconds in a Sonicator XL (Hert System) and spinned at 3000 × g during 10 minutes to eliminate cell debris. The supernatant was centrifuged at 14000 × g for 1 h 30 minutes and the resulting pellet was resuspended with a saline buffer solution and stained with 2% phosphotungstate acid.

### Virus isolation

All nasal swab samples were previously checked for the presence of equine viruses associated with respiratory disease. Equine adenovirus, rhinovirus, arteritis virus, equine herpesvirus 1 and 4, were checked by isolation in cell tissue culture while equine influenza virus was assayed using embryonated eggs. Once it was confirmed that all the samples were negative for the mentioned viruses, the presence of EHV-2 was analysed. Briefly, 0.5 ml of each sample was inoculated onto confluent RK-13 monolayers in 25 cm^2 ^flask (NUNC™, Denmark). After an adsorption period at 37°C for 1 h, the inoculum was removed and cells were maintained at 37°C at an atmosphere of 5% CO_2 _for 7 days with periodic microscopical examination. After this time period, a blind passage was made if CPE was absent. Cell monolayers were thawed and freezed three times and an aliquot of the suspension was passed on to fresh cell culture. The samples were considered negative after the forth-blind passage.

### Nested PCR

The nested PCR used in this study amplifies a 620 bp sequence located upstream of the ORF coding for vIL-10 [27].

Original nasal swab samples were centrifuged for 10 min at 15,000 × g as described elsewhere [27]. Briefly, resulting pellets were diluted in 50 μl digestion buffer (50 mM Tris pH 8.5; 1 mM EDTA; 0.5% Tween 20) plus 1.5 μl Proteinase K (10 mg/ml, Sigma) and incubated at 56°C for 3 h, boiled for 10 min at 95°C to inactivate the proteinase K and the supernatant used for nPCR. PCR conditions were optimised using purified viral DNA from the EHV-2 LK strain obtained as described below. The PCR mix (50 μl) contained 0.2 mM dNTPs, 0.4 μM of each primer [27], 1.5 U Taq polymerase (QIAGEN), 1 × reaction buffer (QIAGEN) and 10 μl of DNA suspension obtained from the original nasal swab sample. The optimal annealing temperature for each of the two primer pairs was 60°C. Cycling was carried out using a Biometra trio-thermblock (Biometra^®^) with 35 amplification cycles consisting of denaturation at 94°C for 30 sec, annealing at 60°C for 30 sec and extension at 72°C for 1 min. For the second round of this nested PCR, 3 μl from the first PCR reaction were amplified with the inner pair of primers using the same cycle as before. Purified viral DNA from the EHV-2 LK strain was used as a PCR positive control.

### Restriction enzyme assay

Viral DNA was obtained from RK-13 cells infected with EHV-2 isolates and also with EHV-2 reference strains H40, LK4, T400 and EHV-5 (P48). Viruses were pelleted from cell culture supernatant by centrifugation through a 30% sucrose cushion at 24,000 × g for 1 h 30 min. Pellets were resuspended with DNA buffer (10 mM MgCl2, 2 mM CaCl2, 200 mM Tris pH 7.4) and treated with RNAse (10 mg/ml) and DNAse I (2 mg/360 μl) at 37°C for 1 h. Subsequently, the pellets were treated with EDTA (0.2 M), 1% sodium dodecyl sulphate and proteinase K (10 mg/ml) (Boehringer, Manheim). Finally, DNA was purified by Genomic-tip 20/G column (QIAGEN, Hilden, Germany) according to the manufacturer's instructions. Aliquots of 200 ng of the obtained purified viral DNA were digested overnight at 37°C with *Eco *RI and *Hind *III. The fragments were separated by electrophoresis through 1% agarose gel and stained with ethidium bromide.

### Statistical analysis

Two (2) ways ANOVA [28] followed by post hoc comparisons (Tukey HSD for unequal N, Spiotvoll/Stoline test) [29] were used.

## List of abbreviations

EHV-2, equine herpesvirus 2; EHV-5, equine herpesvirus 5; E-MEM, Eagle's minimum escential medium; EM, electron microscopy; IIFA indirect immunofluorescence assay; Ab, antibody; PCR, polymerase chain reaction; REA, restriction enzyme assay; NT, neutralization test; CPE, cytopathic effect; vIL-10, viral interleukin 10; ORF, open reading frame.

## Authors' contributions

M.I. Craig carried out the experimental work and drafting of the manuscript. M.E. Barrandeguy carried out the data collection procedure and conceived the study. F.M. Fernández coordinated the project. All authors read and approved the final manuscript.
